# The Association between Neprilysin gene polymorphisms and Alzheimer’s disease in Tibetan population

**DOI:** 10.1002/brb3.2002

**Published:** 2020-12-13

**Authors:** Siwei Chen, Dunzhu Mima, Haiqiang Jin, Qu Dan, Fei Wang, Juan Cai, Lin Shi, Huali Wang, Ailian Du, Ying Tang, Yongan Sun

**Affiliations:** ^1^ Department of Neurology Peking University First Hospital Beijing China; ^2^ Department of Neurology People's Hospital of Tibet Autonomous Region Tibet Autonomous Region China; ^3^ Clinical Laboratory People’s Hospital of Tibet Autonomous Region Tibet Autonomous Region China; ^4^ Department of Neurology Harbin Medical University First Hospital Harbin China; ^5^ Shenzhen BrainNow Research Institute Shenzhen China; ^6^ Beijing Dementia Key Lab National Clinical Research Center for Mental Disorders Peking University Institute of Mental Health (Sixth Hospital) Beijing China; ^7^ Department of Neurology Tongren Hospital Shanghai Jiaotong University School of Medicine Shanghai China

**Keywords:** Alzheimer's disease, genetic polymorphism, neprilysin, risk factor, Tibetan population

## Abstract

**Objectives:**

Alzheimer's disease (AD) is a well‐known neurodegenerative disease, of which the hallmark is the disposition of β‐amyloid (Aβ) in the form of plaque in the brain. Neprilysin (NEP) is the major enzyme to degrade Aβ and prevent accumulation of Aβ. The present study was undertaken to elucidate the correlation between the NEP gene polymorphisms and AD in Chinese Tibetan population.

**Methods:**

Ninety‐nine sporadic AD Tibetan patients and 113 healthy Tibetan controls were enrolled in this study. The genotype frequencies and allele frequencies of multiple NEP gene loci were analyzed using the case–control association analysis.

**Results:**

No significant correlation was found between polymorphisms of NEP gene loci (rs9829757, rs1816558, rs6776185, rs3736187, rs701109, rs989692) and the occurrence of AD in Tibetan population. However, allele C of NEP gene locus (rs701109) and allele T of gene locus (rs3736187) were possible risk factors of male AD patients in Tibetan population.

**Conclusions:**

NEP gene loci (rs701109, rs989692, rs9829757, rs3736187, rs1816558, rs6776185) were polymorphic in Tibetan population. No difference was found between these loci but for that male gender combined with allele C of NEP gene locus (rs701109) and T of gene locus (rs3736187) might be risk factors for AD in Tibet.

## Abstract 2

In this study, single nucleotide polymorphisms of neprilysin (NEP) gene in Tibetan were studied, attempting to elucidate their relevance to the risk of AD. In conclusion, no difference was found between these loci but for that male gender combined with allele C of NEP gene locus (rs701109) and allele T of gene locus (rs3736187) might be risk factors for AD in Tibet.

## 1 INTRODUCTION

Alzheimer's disease (AD) is the most common dementia among the aged population and imposes enormous burden to the family and society. The pathophysiology of AD encompasses amyloid plaques, neurofibrillary tangles, neuronal loss, and astrocyte proliferation (Braak & Braak, 1991; Shao et al., 2017). In particular, the senile plaques (SPs), the compounds of β‐amyloid (Aβ) peptides, are the prominent marker of the AD (Grimm et al., 2015). As one of the amyloid‐degrading enzymes (ADEs), neprilysin (NEP) plays a major role in the degradation process and it accounts for as much as the 50% of the total clearance activity in the brain (Grimm et al., 2015; Saito et al., 2003). Plenty of in vitro and in vivo experiments have demonstrated the activity of NEP as a key factor of AD (Fukami et al., 2002; Guan et al., 2009; Huttenrauch et al., 2015; Iwata et al., 2001; Kanemitsu et al., 2003; Liu et al., 2009, 2010). To date, an array of genetic studies has been reported investigating the potential contribution of NEP (Clarimon et al., 2003; Fu et al., 2009; Helisalmi et al., 2004; Miners et al., 2012; Wang et al., 2016; Wood et al., 2007) and different single nucleotide polymorphisms (SNPs) located in the promoter region of GT repeat, the intron and exons region with increased risk of developing AD. However, there is still no consensus on this potential contribution (Miners et al., 2012; Wang et al., 2016; Zhou et al., 2010). As a special population living in the highest plateau in the world, Tibetan people maintain special lifestyle compared with people from other parts of the world. Whether the SNPs of NEP exist in this population and whether they are associated with AD are still unknown. In this study, SNPs of NEP gene in Tibetan were studied, attempting to elucidate their relevance to the risk of AD.

## 2 MATERIALS AND METHODS

### 2.1 Patients and public involvement

For our participants, a group of 8,268 Tibetan subjects aged over 50 years were recruited from an epidemiological study conducted in Lhasa during year 2011 to 2012. About 3.87% of these people were AD patients. The epidemiological distribution and related risk factors are consistent with the basic laws of AD epidemiology results conducted at home and abroad. The subjects of this study were selected from the subject pool mentioned above, which included 99 sporadic AD patients (43 males and 56 females with a mean age of 68.4 ± 8.3 years) and 113 age‐matched normal controls who had no history of any kind of dementia (42 males and 71 females with a mean age of 67.2 ± 8.6 years). This study was approved by the Ethics Committee of the People's Hospital of Tibet Autonomous Region according to the standards of the Declaration of Helsinki (ID: ME‐TBHP). Informed consents were obtained from all subjects or their lineal relatives in this study.

### 2.2 Standard of diagnosis

All AD participants were diagnosed as probable AD according to NINCDS‐ADRDA criteria (McKhann et al., 1984). Exclusion criteria for AD group included the following: a) other types of dementia; b) recent history of brain trauma or special drug intake; c) alcohol or drug dependence in the past 6 months; and d) history of severe physical disease, other brain organic disease, mental disease, or congenital mental retardation. In the meanwhile, age‐matched normal controls were those who had no manifestation of cognitive impairment and their MMSE grades were more than 26. Those who agreed to provide blood sample and written informed consent were finally recruited.

### 2.3 Genomic DNA extraction and NEP genotyping

Genomic DNA was extracted from peripheral blood by using TIANGEN DNA extraction kit. Six single nucleotide polymorphisms (SNPs) of NEP were selected for screening, and amplification assays were designed for each SNP by Sequenom Assay Design 3.1. All the primers for the 6 loci (rs701109, rs989692, rs9829757, rs3736187, rs1816558, rs6776185) were reported in Table 1. After the examination of MALDI‐TOF (Matrix‐assisted laser desorption/ionization‐time of flight) and a PCR‐based genotyping method, the genotype was obtained by MALDI‐TOF‐MS reaction and Typer 4.0. The genotype frequencies and allele frequencies of multiple NEP gene loci were analyzed using the case–control association analysis.

**TABLE 1:** Primer sequence of NEP gene

SNPs ID	Primers
rs701109	ACGTTGGATGTTGATAGACCTTAGACCCCC
rs989692	ACGTTGGATGCGAATGAACACATGAAGTAG
rs9829757	ACGTTGGATGGTTCAAGACTACACAACTCG
rs3736187	ACGTTGGATGTCTTGTTGCAATGAGTTCCC
rs1816558	ACGTTGGATGGGCCCATGTGCTTTTCTTAC
rs6776185	ACGTTGGATGCTTGAAATTGTAGGACTTC

### 2.4 Statistical analysis

The data were analyzed by SAS 9.4. Differences in sex, age, hypertension, and type 2 diabetes mellitus were compared with chi‐square (χ^2^) test or paired *t* test. Allele frequencies for NEP gene in two groups were calculated and examined by Hardy–Weinberg's equilibrium law. The chi‐square (χ^2^) test and the Fisher exact probability method were used to compare the frequency distributions of NEP genotypes and alleles in the two groups. The odds ratio (OR) of the allelic frequencies of NEP and AD was calculated. All inspection levels are α = 0.05.

## 3 RESULTS

### 3.1 Demographics details

There were no significant statistical differences in gender structure, age, blood pressure history, and type 2 diabetic mellitus history between AD cases and normal controls (*p* > .05). The results are shown in Table 2.

**TABLE 2:** Characteristics of 99 AD patients and 113 controls

Variable	AD group (*n* = 99)	Control group (*n* = 113)	*p* value
gender (male/female)	43/56	42/71	.35
age (year)	68.4 ± 8.3	67.2 ± 8.6	.285
Hypertension	30 (30.30%)	24 (21.24%)	.131
Type 2 Diabetes Mellitus	18 (18.18%)	15 (13.27%)	.325

Note

There was no significant difference in gender, age, history of hypertension, and type 2 diabetic mellitus between AD group and control group (*p* > .05).

### 3.2 Distribution of genotypes and alleles

The frequency distribution of genotypes and alleles in the case and control groups passed the chi‐square test was consistent with Hardy–Weinberg's law of genetic equilibrium, indicating that the gene frequency has reached a genetic equilibrium and has population representativeness. There was some sample loss for not working out the genotype for each locus (5 samples not detected out for rs701109, 6 for rs989692, 6 for rs9829757, 7 for rs3736187, 6 for rs1816558, and 7 for rs6776185). In spite of these sample loss, sensitivity analysis was performed and it had no effect on the final results.

### 3.3 Neprilysin polymorphism

The study revealed that the target NEP gene loci are polymorphic in the Tibetan population. However, there was no significant difference (*p* > .05) in genotype frequency and allele frequency distribution of the 6 SNPs between AD group and control group in the Tibetan population (*p* > .05) (Table 3). However, when stratified by gender, there was a statistically significant difference (*p* < .05) in the frequency distribution of rs701109 and rs3736187 alleles between the AD group and the control group (Table 4 and Table 5). In the case of rs701109, the incidence of carrying C allele for male AD patients was higher (75%) than that of male in the control group (57.5%) (*p* = .02). Meanwhile for rs3736187, comparing to the male control group, the genotype frequency was 2.5% of CC, 32.5% of TC, and 65.0% of TT, and in male AD patients, it was found to be 0 of CC, 12.2% of TC, and 87.8% of TT, respectively (*p* = .03). Besides, male individuals carrying C allele of rs3736187 had lower risk than carrying T allele (6.1% vs. 93.9%, 18.75% vs. 81.25%, *p* = .017).

**TABLE 3:** Genotype and allele frequencies of polymorphisms

	Genotype frequency (%)				Allele frequency (%)			
Locus	Genotype	AD (*n* = 98)	Non‐AD (*n* = 109)	*p* value	Allele	AD (*n* = 98)	Non‐AD (*n* = 109)	*p* value
rs701109	CC	50.00	42.20	.480	C	71.43	66.06	.240
	CT	42.86	47.71		T	28.57	33.94	
	TT	7.14	10.09					

	Genotype	AD (*n* = 97)	Non‐AD (*n* = 109)	*p* value	Allele	AD (*n* = 97)	Non‐AD (*n* = 109)	*p* value
rs989692	CC	54.64	52.29	.943	C	73.71	72.48	.778
	CT	38.14	40.37		T	26.29	27.52	
	TT	7.22	7.34					

	Genotype	AD (*n* = 97)	Non‐AD (*n* = 109)	*p* value	Allele	AD (*n* = 97)	Non‐AD (*N* = 109)	*p* value
rs9829757	CC	54.64	55.96	.964	C	73.20	74.31	.797
	CT	37.11	36.69		T	26.80	25.69	
	TT	8.25	7.34					

	Genotype	AD (*n* = 97)	Non‐AD (*n* = 108)	*p* value	Allele	AD (*n* = 97)	Non‐AD (*n* = 108)	*p* value
rs3736187	CC	0.00	1.85	.501	C	9.79	13.43	.284
	TC	19.59	23.15		T	90.21	86.57	
	TT	80.41	75.00					

	Genotype	AD (*n* = 97)	Non‐AD (*n* = 109)	*p* value	Allele	AD (*n* = 97)	Non‐AD (*n* = 109)	*p* value
rs1816558	GG	14.43	14.68	.844	G	40.21	38.53	.728
	GA	51.55	47.71		A	59.79	61.47	
	AA	34.02	37.61					

	Genotype	AD (*n* = 96)	Non‐AD (*n* = 109)	*p* value	Allele	AD (*n* = 96)	Non‐AD (*n* = 109)	*p* value
rs6776185	GG	2.08	0.00	1.000	G	11.46	9.63	.629
	GA	18.75	19.27		A	88.54	90.37	
	AA	79.17	80.73					

Note

There was no significant difference in genotype frequency and allele frequency distribution of the 6 SNPs between AD group and control group in Tibetan population (*p* > .05).

**TABLE 4:** Comparison of gene frequency distribution of rs701109 between genders

Gender	Group	Cases	Genotype frequency (%)			Allele frequency (%)	
			CC	CT	TT	C	T
Male	AD	42	52.38	45.24	2.38	75.00^a^	25.00^a^
	non‐AD	40	27.50	60.00	12.50	57.50	42.50
*p* value			.0651			.0209	
Female	AD	56	48.21	41.07	10.71	68.75	31.25
	non‐AD	69	50.72	40.58	8.70	71.01	28.99
*p* value			.917			.698	

Note

Genotype frequency between genders: χ^2^ = 2.718, *p* = .257; allele frequency between genders: χ^2^ = 0.575, *p* = .448.

^a^ means the comparison between group AD and controls, *p* < .05.

**TABLE 5:** Comparison of gene frequency distribution of rs3736187 between genders

Gender	Group	Cases	Genotype frequency (%)			Allele frequency (%)	
			CC	TC	TT	C	T
Male	AD	41	0.00^a^	12.20	87.80^a^	6.10	93.90^a^
	non‐AD	40	2.50	32.50	65.00	18.75	81.25
*p* value			.0323			.0171	
Female	AD	56	0.00	25.00	75.00	12.50	87.50
	non‐AD	68	1.47	17.65	80.88	10.29	89.71
*p* value			.3801			.6877	

Note

Genotype frequency between genders: *p* = .934; Allele frequency between genders: χ^2^ = 0.10, *p* = .745.

^a^ means the comparison between group AD and controls, *p* < .05.

## 4 DISCUSSION

Living the highest plateau in the world and with the oxygen level of about 60% of that at the sea level, Tibetan people maintain special lifestyle compared with people from other parts of China. Aside from aging, the level of NEP could decrease due to other cofounding factors such as ischemia and hypoxia (Fisk et al., 2007; Kerridge et al., 2015; Nalivaeva et al., 2012). However, this research contributes to enriching the knowledge of NEP polymorphisms in different ethnicities, which is of particular scientific importance as it is on a population living in the highest altitude.

NEP gene is located on the Chromosome 3 *(3q21‐27)*, spanning more than 80kb and containing 24 exons (Bayes‐Genis et al., 2016; D'Adamio et al., 1989). The expression and catalytic activity are in relevance to age (Zhang et al., 2017). Observation suggested that slight variance on the NEP gene could result in the subtle alternation in the NEP structure or expression, for example, a polymorphism in the 3 UTR region of the NEP gene which is associated with AD in an age‐dependent manner (Clarimon et al., 2003).

To date, several NEP SNPs have been reported to be linked with AD risk, while a number of SNPs were irrelevant to AD risk (Helisalmi et al., 2004). Previous report highlights that the two SNPs (rs989692 and rs373618) may increase the risk of AD development (Helisalmi et al., 2004). A case–control study in Barcelona has displayed that rs701190 was associated with AD in Spanish populations in an age‐dependent manner (Clarimon et al., 2003). For male Tibetan, rs701109 and rs3736187 mutation might add to the risk of AD according to the allele distribution results. But for the whole Tibetan population, these two NEP loci made no significance. This could be due to the difference in sample sizes and races with other studies. Besides, when it comes to the locus rs989692 stratified by gender, the frequency of allele C in AD group and control group had the *p* value of .049, which is a marginal value for the difference test. But for the genotype rs989692 in male individuals, there is no difference between two groups (*p* = .13). Thus, the male individuals carrying allele C from rs989692 elicited higher risk for AD and excluded even though this locus has been reported to have positive result in Finnish population (Helisalmi et al., 2004). More specimens should be added to our study to see whether there is any significant difference in a large sample size for this locus.

In our study, we discussed 6 loci. While there are studies investigating 2 SNPs (Fu et al., 2009) or 7 SNPs (Miners et al., 2012), there are some overlaps for the selection of loci but also remaining different loci, contributing to the different results. A series of studies and meta‐analysis (Gough et al., 2011; Llorca et al., 2008; Ridge et al., 2013; Shao et al., 2017) showed that apolipoprotein E gene (APOE) ε4 is the well‐known risk factor for sporadic AD, including early‐onset AD (EOAD) and late‐onset AD (LOAD). The decrease of NEP expression is associated with APOE ε4 (Gough et al., 2011). Our study has not detected APOE gene in both groups, and there has been no comparison for cases carrying APOE gene in each group, which could lead to different conclusions with other studies. However, APOE ε4 does not affect the distribution of NEP gene polymorphisms (Fu et al., 2009). Bias could also come from other genes that have not been screened such as PS1, PS2, and CLU, which can affect the mechanism of AD development (Baranello et al., 2015; Ridge et al., 2013). In another study, covering 1,475 individuals of two independent Han Chinese case–control cohorts reported that SNP rs1816558 of NEP showed strong linkage to AD risk after adjustment for ε4 allele of the apolipoprotein E gene (APOEε4) and the Bonferroni correction (Wang et al., 2016). It has been shown that no association with AD in Finnish and Tibetan population. Thus, ethnic population prospectively may play a role in the work.

## 5 CONCLUSIONS

NEP gene loci were polymorphic in the Tibetan population. No difference was found between these loci but for the gender combined with allele may be risk factors for AD in Tibetan. Allele C of NEP gene locus (rs701109) and allele T of gene locus (rs3736187) might be risk factors of male AD patients in the Tibetan population. To further complete the NEP polymorphism series study, evidences from more ethnic groups and more participants are needed.

## 6 DATA SHARING STATEMENT

The data that support the findings of this study are available on request from the corresponding author. The data are not publicly available due to privacy or ethical restrictions.

## CONFLICT OF INTEREST

The authors declare that the research was conducted in the absence of any commercial or financial relationships that could be construed as a potential conflict of interest.

## AUTHOR CONTRIBUTIONS

DM, YS, and YT designed the work. DM, QD, and ZP collected the data. FW and JC contributed to the laboratory work and the statistics analysis. SC, HJ, and YS drafted the manuscript. LS, HW, and AD polished the manuscript and helped language editing. All the authors approved the final version of the manuscript.

## Funding information

This work was supported by National Key R&D Program of China (2018YFC1314200) and by Science and technology department of Tibet Autonomous Region (XZ2017ZR‐ZYZ13).

### Peer Review

The peer review history for this article is available at https://publons.com/publon/10.1002/brb3.2002.
